# Marginal Mandibulectomy in Oral Cavity Cancers — Classification and Indications

**DOI:** 10.1007/s13193-024-02102-w

**Published:** 2024-10-11

**Authors:** Sudhir Nair, Hitesh R. Singhavi, Vidula Mestry, Rathan Shetty, Poonam Joshi, Pankaj Chaturvedi

**Affiliations:** 1 Head and Neck Surgery, Department of Surgical Oncology, ACTREC, Homi Bhabha National Institute, Tata Memorial Centre, Navi Mumbai, India; 2 Head and Neck Surgery, Department of Surgical Oncology, Fortis Hospital Mulund, Mumbai, India; 3 Head and Neck Surgery, ACTREC, Tata Memorial Centre, Navi Mumbai, India

**Keywords:** Marginal mandibulectomy, Oral cavity cancers, Segmental mandibulectomy

## Abstract

Squamous cell carcinoma of the lip and oral cavity (OSCC) is a significant global health issue, particularly in low-income countries, with an estimated 390,000 cases detected annually. Although surgery remains the primary treatment option, complex resections are frequently required to attain clear margins. Traditionally, a part of the jaw bone close to the tumour is resected (segmental mandibulectomy) during the surgery. However, marginal mandibulectomy (MM), involving the resection of the mandibular rim while preserving its continuity, offers a less debilitating alternative to segmental mandibulectomy (SM) in selected cases. This review examines marginal mandibulectomy’s oncological safety and efficacy and its current role in managing oral cavity cancers, as indicated by the most recent literature. MM is an effective treatment for tumours, which abut the mandible without bone invasion and provides comparable oncological outcomes to SM, with fewer functional and cosmetic deficits. The authors also propose a classification based on the plane of resection and location within the mandible. We believe this classification will be helpful in reporting the MM series done in various centres in a uniform fashion. However, there is a need for precise surgical planning before doing an MM for obtaining the optimal results.

## Background

Squamous cell carcinoma of the lip and oral cavity (OSCC) accounts for approximately 390,000 cases annually, representing about 2% of the global cancer burden and ranking as the 16th most diagnosed cancer worldwide according to Globocan 2022 [1]. The incidence of these cancers shows significant geographical variation. High incidence rates are observed in South-Central Asia, particularly in India and neighbouring countries, due to the widespread use of tobacco and areca nut in these regions. In contrast, more developed regions such as North America and Europe report lower incidence rates of oral cancer, attributed to better health infrastructure and lower prevalence of risk factors. According to NCCN guidelines, surgery is the standard of care in early oral cavity cancers, while even in advanced stages, surgery followed by adjuvant treatment remains the mainstay [2]. In surgical management, the goal is to remove the tumour with at least a 5 mm margin free of cancer cells. Studies have demonstrated significant improvement in both local and regional control when margins exceed 5 mm, a factor influenced by the surgeon that determines patient survival [3]. Positive margins adversely affect survival and necessitate postoperative concurrent chemo-radiotherapy.

The oral cavity is divided into seven subsites: lip mucosa, gingiva, alveolus, floor of mouth, tongue, hard palate, and retromolar trigone. The floor of the mouth, lower alveolus, and retromolar trigone are subsites close to the mandible. When disease involves these sites, achieving clear margins may necessitate mandible resection, even if the bone is not anatomically involved. Loss of mandibular continuity can result in significant functional and cosmetic issues for patients. To mitigate these problems, bony reconstruction of the mandible, often achieved through microvascular free flap reconstructions like the fibula flap, is essential. However, when the bone is not involved by the tumour but the resection margin can be close to the mandible, a marginal mandibulectomy (rim resection of the mandible) can be considered to achieve clear margins. This type of resection maintains the continuity of the mandible, thereby preserving occlusion without compromising oncological safety. This review aims to assess the current literature on the oncological safety, complications, and current role of marginal mandibulectomy in the management of oral cavity cancers.

## Methods and Materials

This review is based on the most recent publications indexed in PubMed and Google Scholar on marginal mandibulectomy for oral cavity cancers. There were no specific inclusion or exclusion criteria applied when selecting articles for inclusion in this study. The review identifies the most pertinent articles and discusses the key findings from each study, focusing on aspects that are considered significant for the management of this condition. Those articles published between 2010 and 2024 are considered to contain the most up-to-date information about this procedure.

Previously, it was thought that the lymphatic system in the mandible was responsible for the spread of cancer through the mandible. Hence, squamous cell carcinomas (SCCs) on the floor of the mouth (FOM) or in the oral tongue close to the mandible were removed along with an adjacent portion of the mandible. Unfortunately, this approach resulted in significant functional and aesthetic challenges for patients. Later, Marchetta and Carter found that cancer spreads directly to the mandible rather than through the lymphatic system. It was thus possible to consider preserving a portion of the mandible rather than removing a large segment. The concept of marginal mandibulectomy thus became an essential tool for the management of cancers in these sites.

## Marginal Mandibulectomy — Technique

In simple terms, marginal mandibulectomy (MM) involves the removal of a tumour along with a rim of the adjacent mandible bone while preserving the continuity of the mandible. This technique had been in use for over 50 years (Shah et al.) but became more popular and well-defined after the 1990s [4]. Conventionally, the surgical management of oral cancer involving or lying adjacent to the lower jaw was excised in continuity with the segment of the mandible (segmental mandibulectomy). However, such radical procedures resulted in significant functional and cosmetic issues. Marginal mandibulectomy offers a less debilitating alternative for cases where the cancer is close to but not invading the mandible.

The common indications for marginal mandibulectomy include.

- Tumours of the oral cavity abutting the mandible without invading it.
- Very superficial lesions involving the periosteum or causing minor cortical erosion of the mandible.

Marginal mandibulectomy should be avoided when there is invasion of the mandible or in large tumours with para-mandibular spread.

## Classification: Types of Marginal Mandibulectomies (MM) (Figs. 1 and 2)

**Fig. 1:**
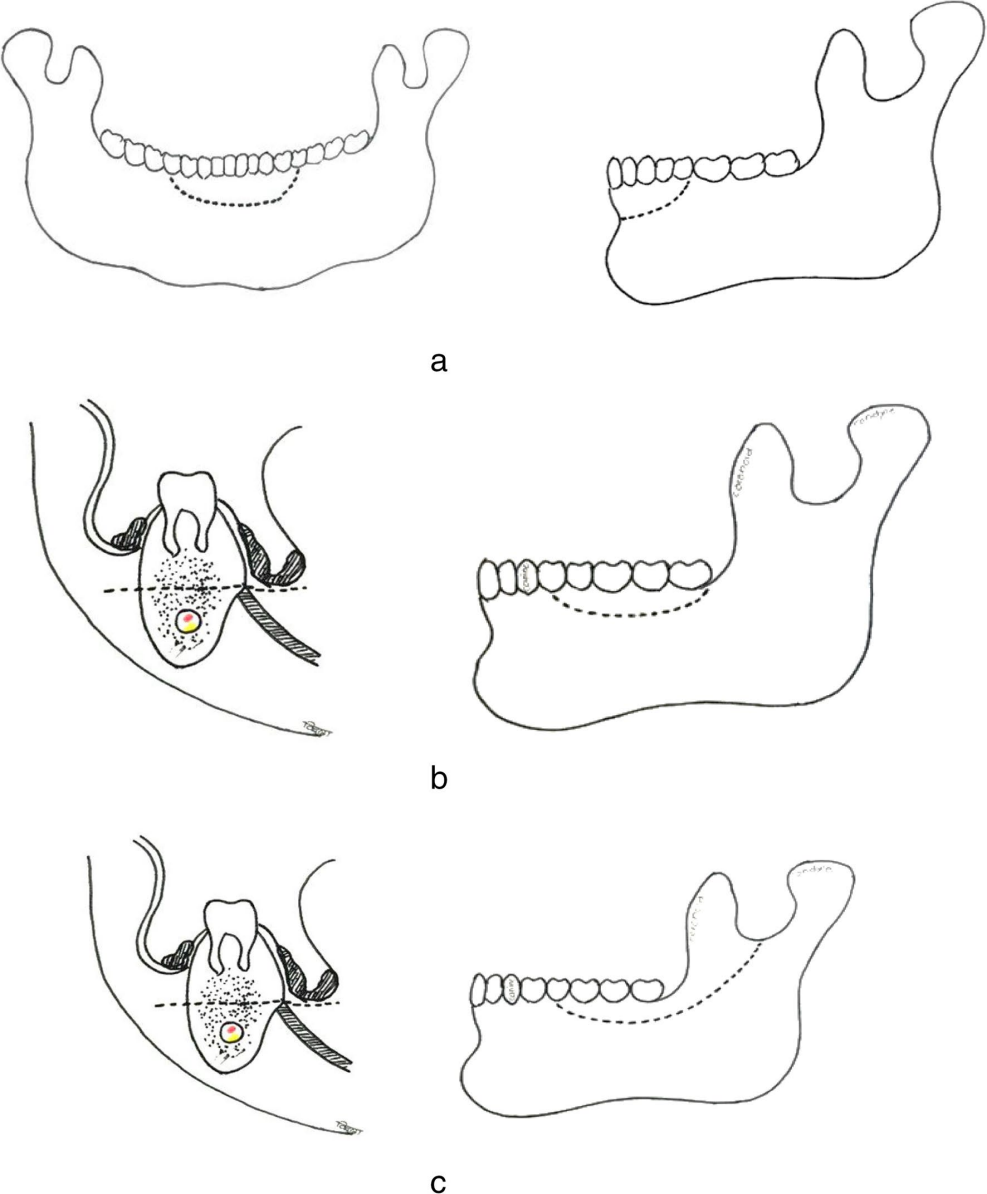
Type 1 marginal mandibulectomy. Type 1**a**: anterior marginal mandibulectomy. The dashed lines depict the proposed bone resection in the anterior portion of the mandible, sparing the inferior border. The left image shows the occlusal view and the right image shows the lateral view of the mandible. Type 1**b**: marginal mandibulectomy on the lateral aspect (without removal of the coronoid process). The left image provides an axial cross-sectional view, depicting the lesion location and the extent of resection, while the right image shows a lateral view of the mandible with the planned marginal resection. Type 1**c**: marginal mandibulectomy on the lateral aspect (with the removal of the coronoid process). The left image is an axial cross-sectional view showing the lesion and the planned resection area, while the right image presents a lateral view of the mandible, highlighting the resection of the alveolar ridge and the coronoid process

**Fig. 2:**
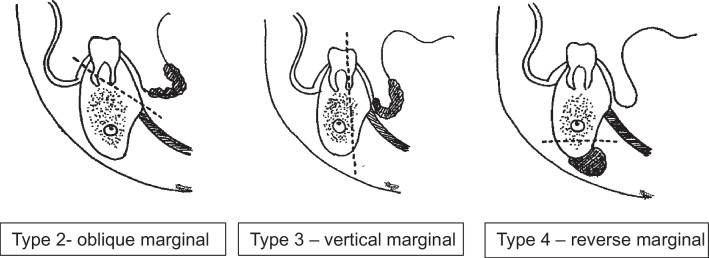
Marginal mandibulectomy (oblique, vertical, reverse)

MMs are traditionally classified based on the location or plane of resection. Accordingly, it can be Anterior MM or Posterior MM based on the location anterior or posterior to the canine, respectively. However, we observe inconsistencies among authors in describing the precise locations of the MMs. Similarly, as per the plane of resection, MMs can be horizontal, vertical, or oblique (Shaha). Considering the commonly performed marginal mandibulectomies, we propose a uniform classification for describing different types of mandibulectomies. It is as follows:

- Type I: Horizontal marginal mandibulectomy.
  - Here, the mandibular rim is resected horizontally posterior to the molar to the coronoid process, and the coronoid process may or may not be removed. However, the continuity of the mandible is maintained, and a minimum height of 1 cm is kept at the residual mandible for its strength. This type of mandibulectomy is performed for small primary lesion located in the posterior locations like retromolar trigone. Can be further subdivided into three groups.
    - Type Ia: Anterior marginal mandibulectomy (any segment anterior to the first molar)
    - Type Ib: Coronoid sparing posterior marginal mandibulectomy (posterior to the 2nd premolar)
    - Type Ic: Posterior marginal mandibulectomy with coronoidectomy
- Type II: Oblique marginal mandibulectomy.
  - Performed for tongue or floor of mouth lesions close to the mandible when mucoperiosteal stripping may not yield adequate margin. The lesion also is away from the mylohyoid.
- Type III: Vertical marginal mandibulcetomy on lingual side.
  - This type involves a vertical resection of the lingual plate of the mandible, focusing on the internal aspect of the mandible. Performed for same indications above but when suspecting a deeper tumour. However, not routinely performed as it is technically challenging and can negatively affect the strength of the residual mandible.
- Type IV: Reverse marginal mandibulectomy.
  - Resection of the lower rim of the mandible, when cervical lymph nodes are adherent to the mandible without cortical erosion.

### Indications for Marginal Mandibulectomy

Based on the above classification, we shall review the role of marginal mandibulectomy for surgical management of oral cancers in different subsites.

### Cancers of the Retromolar Trigone (RMT)

The retromolar trigone is a small, triangular area in the oral cavity located just behind the last molar on the lower jaw. The anterior edge (posterior edge) of the last molar forms its anterior border, while the anterior edge of the ascending ramus of the mandible forms its posterior border. This anatomical region plays a significant role in oral oncology due to its proximity to critical structures such as the mandible, muscles of mastication, and neurovascular bundles. The RMT is challenging to visualize and palpate, especially in patients with limited mouth opening, necessitating the use of radiological evaluations like Contrast-enhanced CT scans. When bony invasion is present, a combined modality treatment is typically recommended, involving surgery (segmental mandibulectomy) followed by postoperative adjuvant radiotherapy (PORT) or concurrent chemo-radiotherapy (POCRT). However, there is some debate regarding the management of smaller lesions in the RMT that do not have bone invasion in scans. Both primary radiotherapy (RT) and primary surgery have shown similar outcomes when bone invasion is absent.

In cases where surgery is the primary treatment choice, some surgeons advocate a more conservative approach, like a posterior marginal mandibulectomy (Type Ib/Ic MM), while others recommend a segmental mandibulectomy. Studies have shown, however, that a Type I MM provides an equivalent treatment outcome to a segmental mandibulectomy while preserving the mandible’s functional integrity (Pathak). Pathak et al. analysed the records of 130 consecutive retromolar trigone cancer patients who underwent marginal or segmental mandibulectomy at a tertiary care centre [5]. Although patients undergoing marginal mandibulectomy had a higher recurrence rate than segmental mandibulectomy (19% vs. 6%), the difference was not statistically significant. Moreover, a subsequent segmental mandibulectomy could salvage 67% of recurrences after a marginal mandibulectomy. This study concluded that segmental mandibular resection should be reserved for more invasive tumours. Additionally, a Brazilian study examined the recurrence and survival rates of patients with retromolar and advanced tonsil tumours with no invasion of the mandible treated between 1994 and 2001 [6]. There was no significant difference between marginal and segmental mandibulectomy regarding recurrence rates or overall survival (55% versus 45%). However, this cross-sectional study consisted of just 20 and 22 patients each in each arm, resulting in a small sample size. We also reported (Nair et al.) a series of 98 cases, where 56 patients underwent Type Ia, and 42 underwent Type I b/c marginal mandibulectomy, respectively. We observed no significant change in the local recurrence rate between anterior marginal or posterior marginal mandibulectomy [7].

### Lower Gingivobuccal Sulcus Tumours

In 1988, McGregor et al. emphasised the occlusal surface, especially in non-irradiated mandibles as the root of entry for tumour from the floor of the mouth [8]. Later studies by Brown et al. [9] found no preferential entry of tumours through the periodontal membrane but noticed direct entry of the tumour at the point of abutment for both dentate and edentulous mandibles. Considering these points, larger and deeper tumours require segmental resection, whereas for smaller tumours, there is a need to consider the direct point of entry, which can be the closest border of the tumour with the mandible. Multiple studies have shown the oncological safety of marginal mandibulectomy especially when there is no cortical bone erosion or marrow involvement. For alveolar disease without bone erosion, a Type I marginal mandibulectomy with wide local excision should be sufficient.

### Floor of Mouth and Tongue Cancers

Patterns of invasion and spread play an important role in the way the cancer spreads and the anatomical structure it will involve according to subsite. In the floor of mouth mucosa, the important anatomical barriers are the mandible laterally, mylohyoid muscle inferiorly, and the least path of resistance is the tongue medially. Thus, usually, FOM tumours abut the mandible rather than involving it directly. Often segmental mandibulectomy is performed to achieve clear margins. However, an oblique marginal mandibulectomy (Type II) or a vertical marginal mandibulectomy (Type III) can clear the disease that extends to the lingual aspect of the lower jaw, without compromising the stability or its continuity.

## Conclusion

Marginal mandibulectomy (MM) is a valuable technique for managing oral cavity cancers abutting the mandible without bone invasion. Studies indicate that MM offers comparable oncological outcomes to segmental mandibulectomy (SM) with less morbidity in selected cases. MM provides effective local control and survival rates, making it a preferable alternative for non-invasive tumours. Proper classification and surgical planning, along with periosteum preservation, are crucial to minimizing complications like osteoradionecrosis. With improvements in reconstruction and prosthetic rehabilitation, Marginal mandibulectomy is a good alternative and less complex surgical procedure when compared to segmental mandibulectomy.
